# Copying hierarchical leaders’ voices? Acoustic plasticity in female Japanese macaques

**DOI:** 10.1038/srep21289

**Published:** 2016-02-16

**Authors:** Alban Lemasson, Ronan Jubin, Nobuo Masataka, Malgorzata Arlet

**Affiliations:** 1 Université de Rennes 1, Ethologie animale et humaine – C.N.R.S., Rennes, France; 2 Primate Research Institute, Kyoto University, Japan; 3 Indian Institute of Science Education Research, School of Biology, Evolutionary Ecology Group, Trivandrum, India

## Abstract

It has been historically claimed that call production in nonhuman primates has been shaped by genetic factors, although, recently socially-guided plasticity and cortical control during vocal exchanges have been observed. In humans, context-dependent vocal convergence with relatives, friends or leaders’ voices can be found. Comparative studies with monkeys and apes presenting tolerant social organizations have demonstrated that affiliative bonding is the determining factor of convergence. We tested whether vocal copying could also exist in a primate species with a despotic social organization. We compared the degree of inter-individual similarity of contact calls in two groups of Japanese macaques as a function of age, dominance rank, maternal kin and affiliative bonds. We found a positive relationship between dyadic acoustic similarity and female rank differences. Since most call exchanges were initiated by dominant females and since this species is known for the ability of responders to acoustically match initiators’ calls, we conclude that high social status may motivate vocal convergence in this despotic society. Accordingly, intra-individual comparisons showed that isolated calls were more stereotyped than exchanged calls, and that dominants had more stereotyped voices than subordinates. This opens new lines of research with regard to social motivation guiding acoustic plasticity in primates.

Several behavioural and cognitive parallels between human and non-human primates have been raised (e.g. social organization, empathy, tool use, hunting, self-recognition among many others^1^) and are widely accepted by the multidisciplinary scientific community. However, among primates, flexible vocal communication has long been considered a feature unique to humans^2^,^3^. This is notably because humans have a socially-guided language development with intentional verbal utterances, whereas non-human primates have been found to produce calls that are strongly influenced by genetic factors, and their acoustic variations are explained by the changes only in maturation or arousal state^4^. However, during the last two decades, this traditional dichotomy has been strongly challenged. For example, parallels with humans have been published on monkeys and apes concerning rules of vocal interactions^5^,^6^,^7^, audience effects on vocal behavior^8^,^9^,^10^, call innovation^11^,^12^,^13^, dialect^14^, prosody^15^, referentiality^16^,^17^ as well as sound combinatorial rules^18^,^19^,^20^. Recent neurobiological studies have also shown context-dependent voluntary brain-motor vocal control, particularly active during social interactions^21^,^22^.

Human language is above all a social act and acoustic plasticity is an important feature at different levels. Of course plasticity enables acquisition of new words through learning. But plasticity can also be used to advertise social bonds and facilitate social integration. This context- and audience-dependent inter-individual acoustic convergence (or divergence) is a social phenomenon known as vocal accommodation in humans, facilitating social integration^23^. For example, vocal convergence based on social status (i.e. professional interviews) or affinity (e.g. college roommate) of interlocutors has been described in humans^24^,^25^.

Dyadic and group vocal convergence seems to be an important ability shared by many social species (songbirds and dolphins^26^, whales^27^, bats^28^, goats^29^, gazelles^30^, elephants^31^). It seems that nonhuman primates have not been exceptions and that the social bond between the converging partners is of prime importance. Vocal copying has been shown in monogamous primate species, (e.g. between pair mates in pygmy marmosets^32^) and between mothers and daughters in agile gibbons^33^. Vocal copying has also been found in species forming multifemale one-male groups, e.g. between strongly socially bonded females in Campbell’s monkeys^34^,^35^, and in species forming multifemale multimale groups, e.g. between affiliated males and females in bonobos^36^. The question remains as to open whether affiliative bonding is the only determining factor driving vocal convergence, or if, the position in the dominance hierarchy can also play a role as in some human contexts.

Macaques are particularly interesting for investigating this problem because they are a clade in which all species form multimale multifemale groups but have strong variations in their social organization^37^. In some species, so-called despotic and hierarchical (e.g. Japanese macaques, rhesus macaques) aggression is frequent and intense, conflicts are mainly unidirectional and reconciliation between opponents is rare^38^. In these species, dominance hierarchies and kin relatedness are two determining factors of social life^39^,^40^. By contrast, some species, so-called tolerant and egalitarian (e.g. Tonkean macaques, crested macaques), rarely show severe aggression, bidirectional conflicts with counter attack are accepted, post-conflict reconciliation is systematic, and dominance hierarchies or kin-relatedness play a minor role^41^,^42^. The characteristics of the social organization clearly co-vary with the communicative repertoire of facial expressions^38^. Hence, one could also expect that social relationships, notably hierarchical bonds, would impact the acoustic structure of vocal signals in macaques. Moreover, abilities in acoustic matching of frequency and temporal parameters during contact call exchanges (i.e. responders acoustically match initiators) have been evidenced in female Japanese macaques^43^, while their social motivation in copying remains an open question.

In this study, we investigated to what degree the inter-individual acoustic variation in female Japanese macaques’ calls could be explained by social bonds (affinity: based on grooming and spatial proximity; dominance rank) and genetic factors (age difference and maternal kin relatedness). We tested the relative importance of all these factors on dyadic acoustic similarity in two captive groups.

## Results

**Figure 1:**
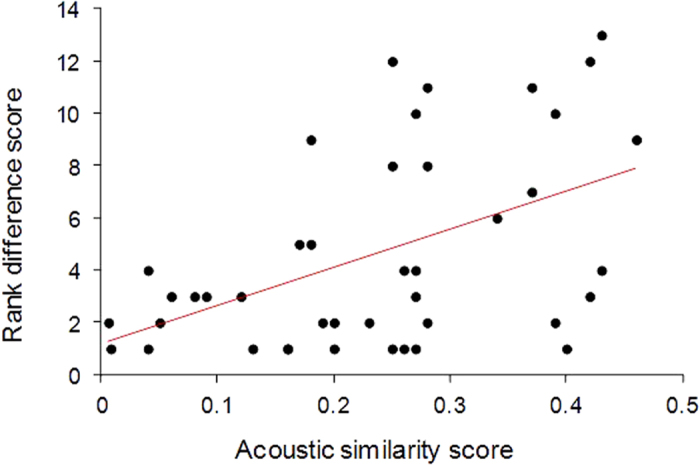
Influence of the dominance rank difference between pairs of adult females on the acoustic similarity of their contact calls.

We found that age difference, maternal kinship and all social affinity scores (spatial proximity, grooming frequency and duration) did not influence inter-individual acoustic similarity of contact calls (Table 1). However, rank difference was a determining factor explaining acoustic similarities (Table 1), with higher acoustic similarities between females who were the more distant in the dominance hierarchy (Fig. 1). The positive correlation between rank difference and acoustic similarity was found in both - Wak and Tak groups (Mantel tests: Wak observ. values 0.553, P = 0.003 and Tak observ. values 0.750, P = 0.001).

Rank position influenced the ordering of callers within vocal interactions, as females initiated more call exchanges while being dominant (mean ± SD = 6.9 ± 6.2) over the respondent than when being subordinate (mean ± SD = 3.5 ± 6.3) to the respondent (Wilcoxon test: N = 14, T = 11, Z = 2.197, P = 0.028). However, importantly, there was no significant difference in calling rate (per focal) between dominant and sub-dominant females (GLMs: df = 1, F = 1.03, P = 0.32), or between all females (df = 15, F = 0.75, P = 0.7).

**Figure 2:**
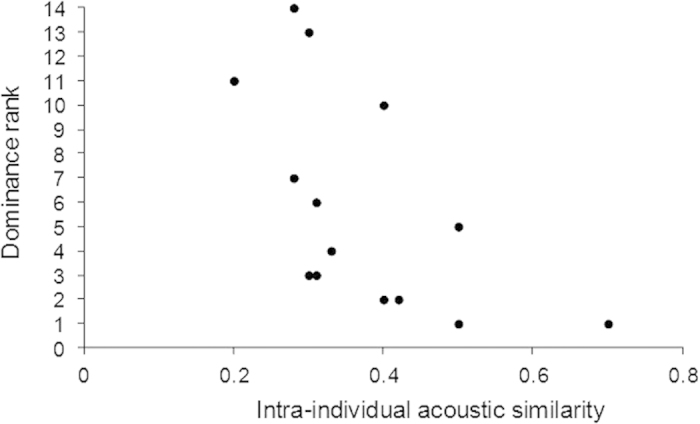
Variation of intra-individual acoustic similarity among females of different ranks in two captive groups of Japanese macaques (N = 14).

**Figure 3:**
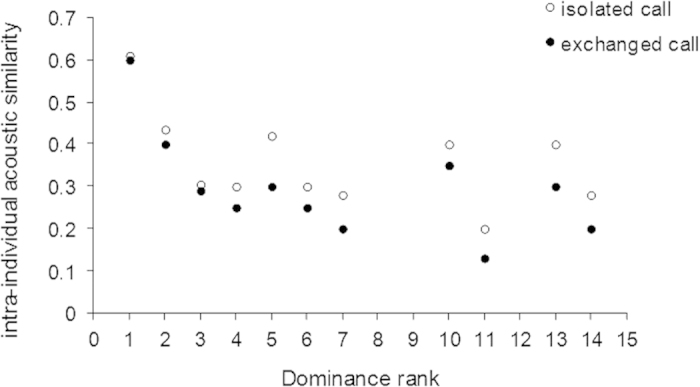
Variation of intra-individual acoustic similarity in isolated versus exchanged calls among females of different ranks in two captive groups of Japanese macaques (N = 14).

Finally, by running intra-individual acoustic comparisons, we found that dominant females produced contact calls that were more acoustically stereotyped that the calls of sub-dominant females which were thus more varied (GLMs: N = 14 females, df = 1, F = 180.5, P < 0.001; Fig. 2). Moreover, overall, isolated calls were more acoustically stereotyped than exchanged calls (N = 14 females, df = 1, F = 43.08, P < 0.001; Fig. 3).

## Discussion

Our results showed that socially-determined vocal convergence also exists in Japanese macaques. As opposed to more tolerant primate species, vocal copying may be driven by the position in the dominance hierarchy rather than by social affinities. Rank difference was the only measured parameter which could predict vocal resemblance between adult females from two captive groups, i.e. the more distant in the hierarchy, the more acoustically similar. Since the position in the hierarchy also predicted the ordering of callers within vocal exchanges, with more initiations by dominant females, and as female Japanese macaques are already known to match the acoustic structure of the call they are responding to^43^, we believe that subordinate females tend to copy leaders’ voices.

Of course, we cannot totally rule out the reversed causal explanation, i.e. already-established call variations might be driving (or at least affecting) the structure of social networks including dominance hierarchies. Another alternative hypothesis that cannot be totally ruled out is that females close to each other in rank may have diverged their calls. However, we believe that our complementary findings, based on intra-individual acoustic comparisons, which demonstrate that dominant females have more stereotyped voices than subordinate ones, and that exchanged calls are more variable than isolated calls, support the idea that subordinate adjust their voices contextually.

This study first confirms the limit of using the genetic background as an explanation for the variation in vocal production in nonhuman primates. If it is clear that the vocal repertoire is globally determined by genetic factors^4^, fine acoustic modifications are still possible throughout adult life^35^. The strong weight of genetic factors of the vocal repertoire in Japanese macaques has been demonstrated earlier with cross-fostering experiments^44^. But the possibility to adjust voice structure to context has also been demonstrated (motherese-like communication with infants^45^, acoustic matching within exchanges^38^ and dialectal acoustic variations^46^).

Moreover, our study highlights how social factors may impact this acoustic refinement, notably in adult nonhuman primates. One well known example is Campbell’s monkeys where previous studies, in both captive^34^ and wild populations^35^, showed that vocal copying was independent of kin relatedness, maturation (age), position in the dominance hierarchy but depended on affinity (based on spatial proximity and grooming^35^). Here, age and maternal kin also do not play a role but dominance hierarchy does while affinity does not. A recent study on rhesus macaques has shown that paternal kinship can shape acoustic structures^47^, this still needs to be investigated in Japanese macaques.

Campbell’s monkeys and Japanese macaques may show different social motivations of vocal copying because the former is tolerant and the latter is despotic. Previous descriptions have been done to show the clear opposition of the two social organization systems in macaques and baboons on the one hand and guenons on the other^48^,^49^. Macaque and baboon social networks are generally more structured around dominance hierarchies than are guenons who have rare agonistic encounters and discrete hierarchies. In line with that, Fisher and colleagues^50^ found that baboon loud calls advertise male dominance rank in a flexible way. Copying leaders is also found in other mammals. Younger peripheral males imitate vocal types of older more successful breeders in elephant seals who form societies with high levels of inter-male competition^51^.

In sum, our study supports the idea of considering acoustic copying as a social strategy^23^, a phenomenon proposed for songbirds, marine mammals, nonhuman and human primates. It make sense that these vocal signatures are usually found in contact calls, which are the most varied (intra- and inter-individual) call types of the repertoire and which are associated to functions with a high social value^52^. Copying social affiliates or leaders may then depends on the social needs of the callers. Hence, there is now a need for more comparative studies with an evolutionary perspective, especially focusing on animal taxa with closely related species with varied social styles such as macaques.

## Methods

### Study areas and subjects

Data were collected from February till August 2005 at the Primate Research Institute (Inuyama, Kyoto University). We conducted behavioural observations and acoustic recordings in two captive groups of Japanese macaques (Wakasa: WAK, and Takahama: TAK). WAK/TAK groups were respectively composed of 10/17 adult males, 17/28 adult females and 4/8 offspring. All individuals were captive born and individually identified, with known ages, maternal kin relationships and dominance ranks. Female ranks were taken from another study based on observations done at the same period^53^.

Based on the 825 dyadic agonistic interactions recorded between 29 focal females, including nonphysical threats (e.g., facial displays), approach–avoids (i.e., moving away from another who is approaching), supplants (i.e., taking the place of another), physical contact (e.g., biting, tail-pulling, and pushing), and chases (i.e., aggressively pursuing another), we constructed dominance matrices for each group, with rank order determined by minimizing the number of reversals against the hierarchy (i.e., interactions below the diagonal). The Landau indices in Wakasa and Takahama groups were respectively h’ = 0.57 (p = 0.002) and h’ = 0.53 (p < 0.001), which indicates a linear dominance hierarchy in both groups. Here, we randomly selected a subset of 14 adult females (seven per group, Table 2).

Outdoor enclosures differed in the two groups. The WAK group was housed in a larger (4600 m^2^) and visually dense (forested) enclosure, while TAK group was housed in a smaller (960 m^2^) and visually open enclosure enriched with several metal perches and shelters. Animals were fed twice a day with monkey chow and sweet potatoes. Water was made available *ad libitum*.

All animal care and data collection protocols were consistent with the Guide for the Care and Use of Laboratory Primates and were approved by the Institutional Animal Care and Use Committee of the Primate Research Institute, Kyoto University.

### Behavioural observations and analysis of social bonds

The two groups were observed twice a day (morning and afternoon), for 90 minutes each session, during which 10-min focal samples were performed on adult females in a random order. Focal sampling consisted in recording the duration of allogrooming interactions and spatial proximities (defined as less than 1 m), the number of agonistic interactions (i.e. chases, approach avoids, supplants, bites, pushes, facial threats) as well as the number of contact call exchanges between females (defined as two, or more, individuals calling in a row with their respecting coos trailing each other with less than 2 s)^43^.

The two groups were observed twice a day (morning and afternoon), for 90 minutes each session, during which 10-minute focal samples were performed on adult females in a random order^54^. Focal sampling consisted of collecting the number of occurrences and durations of allogrooming interactions, the durations of spatial proximities (defined as less than 1 meter) and the number of occurrences of contact call exchanges between the study females. In total, we conducted 451 focal observations (105.5 hrs of contact time), with an average of 36.6 +/− 0.5 S.E. focal samples per female.

Based on the affiliative data of 14 females (7 per group), their intra-group dyadic spatial proximity, grooming scores and aggression were calculated for each of 42 (21 per group ×2 groups) pairs of adult females (i.e. total frequency or duration divided by focal time). Based on the vocal exchanges data (N = 281 vocal interactions), we counted the number of times the two first callers of the vocal interaction were ordered going up or down the dominance hierarchy.

### Acoustic recording and analysis

**Figure 4:**
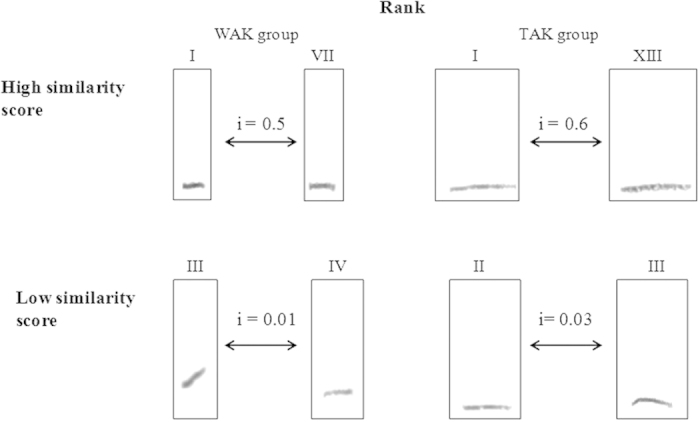
Example of pairs of contact calls and corresponding similarity indices.

Acoustic recordings were simultaneously performed with an ECM-672 Sony© directional microphone connected to a TCD-D100 Sony© DAT recorder (.wav format, sampling rate: 48 kHz, resolution: 16 bit). Contact calls (so-called coos^43^) were individualized into separate audio files (for exchanged and non-exchanged – i.e. isolated - calls separately) using Sound Forge 5.0. An average of 24.07 +/− 0.9 S.E. calls per female was sampled, presenting good acoustic quality (no call or noise overlap and no echo) for the subsequent analysis. Using ANA software (Richard 1991), acoustic similarity indices were calculated by comparing the shape of the frequency modulations of the different calls (for details on previous usage of this measure see: Campbell’s monkeys^34^,^35^, Diana monkeys^55^, agile gibbon^33^). The comparison was based only on the fundamental frequency pattern and not the harmonics. A dyadic acoustic similarity score was obtained, for each pair of females (AB) within both groups, by averaging all the similarity indices obtained from the comparison of each A’s call with each B’s call. The similarity indices were based on pixel by pixel comparisons between pairs of spectrograms. Each pixel was associated with a grey value ranging from 0 to 255. If one or both compared pixels had a zero grey value, a score of “0” was given. If the two compared pixels differed by less than 16 in their grey values, a score of “2” was given. All other combinations were given a score of “1”. The total of all scores was then divided by the total number of pixels in both spectrograms yielding a grey value superior to zero, in order to generate a similarity index which ranged between 0 and 1. The algorithm then carried out the same operation for all possible superpositions by comparing spectrograms along the time axis, which generated similarity indices for each temporal position. Once all temporal positions were compared, the algorithm determined the highest similarity index for the two spectrograms. Examples of comparisons are illustrated in Fig. 4.

### Statistical analysis

We performed most analyses using the statistical software Statistica. We used General Linear Models (GLMs) to test for the impact of social and genetic factors on contact call structures. We tested the influence of spatial proximity, grooming duration and frequency scores, aggression (per focal), maternal kinship (binary: kin or nonkin), dominance rank and age differences, and group (independent variables) with acoustic similarity scores (dependent variable). To investigate further, we run Mantel tests of association to compare a dominance hierarchy matrix with an acoustic similarity matrix (with statistical software R^56^). To make the matrices comparable, both factors measured (similarity index and dominance) needed to be measured in a consistent way. Therefore, we adjusted the rank difference between two females by dividing it by the total number of females in their group.

In order to test for the impact of dominance rank on vocal exchange directions, we compared, using a Wilcoxon matched-pair signed-rank test, the number of times each of our 14 study subjects initiated a vocal interaction while being dominant *vs* subordinate to the respondent. We also investigated the possible relation between calling rate and the dominance rank using GLMs. To investigate the intra-individual acoustic variability, we used GLMs with weighting variable (number of all contact calls), with rank and calling type (isolated or exchanged) as independent variables and with acoustic similarity scores as a dependent variable.

## Additional Information

**How to cite this article**: Lemasson, A. *et al.* Copying hierarchical leaders’ voices? Acoustic plasticity in female Japanese macaques. *Sci. Rep.* **6**, 21289; doi: 10.1038/srep21289 (2016).

## Figures and Tables

**Table 1:** Relation between socio-genetic factors and acoustic similarity (GLMs, P values: likelihood ratio tests; N = 42 pairs of females).

Effect	SS	F	P
Age difference	0.001	0.11	0.74
Rank difference	0.06	8.2	0.007
Maternal kinship	0.003	0.37	0.55
Group	0.02	2.49	0.12
Spatial proximity	0.001	0.01	0.92
Grooming time	0.01	1.22	0.28
Grooming rate	0.002	0.2	0.66
Aggression	0.001	0.01	0.92

**Table 2:** Characteristics of the fourteen females studied.

Female	Group	Age	Matriline
Hasi	WAK	10	1 (mother)
Mina	WAK	4	1 (daughter)
Mini	WAK	6	1 (daughter)
Nira	WAK	6	1 (sister of mother)
Rumi	WAK	17	2 (mother)
Mila	WAK	9	2 (daughter)
Reka	WAK	14	2 (sister of mother)
Yuki	TAK	21	3 (mother)
Kin	TAK	15	3 (daughter)
Tsuyu	TAK	21	3 (sister of mother)
Take	TAK	17	4 (mother)
Iwa	TAK	11	4 (daughter)
Tani	TAK	24	5 (mother)
Ume	TAK	8	5 (daughter)
